# Melatonin alleviates sodium sulfite-induced osteoporosis in mice via suppression of the ferroptosis pathway

**DOI:** 10.1007/s10495-025-02135-8

**Published:** 2025-07-18

**Authors:** Qiuping He, Lei Xie, Haining Peng, Xiao Xiao, Tengbo Yu

**Affiliations:** 1 Department of Orthopedic Surgery, Qingdao Municipal Hospital, Qingdao University, Qingdao, 266000 China; 2 Central Laboratories, Qingdao Municipal Hospital, University of Health and Rehabilitation Sciences, Qingdao, 266000 China; 3 Department of Orthopedic Surgery, Qingdao Municipal Hospital, University of Health and Rehabilitation Sciences, Qingdao, 266000 China

**Keywords:** Sodium sulfite, Osteoporosis, Ferroptosis, Melatonin, MC3T3

## Abstract

Sodium sulfite (SS) is a common food additive that is widely absorbed and distributed throughout the body, but its excessive intake has been linked to adverse health effects. Here, we investigate the impact of chronic SS exposure on bone tissue and the underlying mechanisms. Using a mouse model, we demonstrate that prolonged SS exposure induces significant bone loss, which correlates with alterations in ferroptosis-related markers. In vitro, SS exposure activates ferroptosis, which is characterized by elevated reactive oxygen species levels and impaired osteogenic differentiation in MC3T3 cells. Notably, melatonin, a potent endogenous antioxidant, mitigates SS-induced oxidative stress, inhibits ferroptosis, restores osteoblast function, and alleviates bone loss in mice. These findings highlight ferroptosis as a critical contributor to SS-induced osteoporosis and identify melatonin as a promising therapeutic agent for its prevention and treatment.

**Supplementary Information:**

The online version contains supplementary material available at 10.1007/s10495-025-02135-8.

## Introduction

Sodium sulfite (SS) is a widely utilized food additive approved by the US Food and Drug Administration for its antimicrobial properties, effectively inhibiting the growth of molds, yeasts, and bacteria [1]. Upon ingestion, SS is absorbed through the gastrointestinal tract and distributed to various tissues, including the liver and bones [2]. In addition to dietary exposure, SS is endogenously produced during the metabolism of sulfur-containing amino acids and certain drugs [3]. Despite its widespread use, excessive exposure to SS has been implicated in adverse health effects [4, 5], including hepatocyte apoptosis, mast cell damage, and hypersensitivity reactions, such as asthma [6]. The broader health implications of prolonged SS exposure remain poorly understood and warrant further investigation.

Osteoporosis is the most prevalent metabolic bone disease, characterized by reduced bone mass, compromised microarchitecture, and increased fracture risk [7]. Globally, over 9 million osteoporotic fractures occur annually, posing significant health and socioeconomic burdens [8]. While factors such as aging, hormonal changes, and lifestyle are well-established contributors to OP, the potential role of environmental and dietary factors, such as excessive SS exposure, in its pathogenesis is largely unexplored.

Ferroptosis, a recently identified form of iron-dependent regulated cell death [9], is distinguished by its reliance on iron accumulation and lipid peroxidation. This unique pathway is marked by reduced expression of glutathione peroxidase 4 (GPX4) and glutathione depletion [10]. Ferroptosis has been implicated in a spectrum of diseases, including cancer, cardiomyopathy, and multiple degenerative diseases [11–14]. Emerging evidence suggests that ferroptosis also contributes to bone-related disorders, including osteoarthritis, glucocorticoid-induced osteoporosis, and rheumatoid arthritis [15, 16]. However, its role in the context of excessive SS-induced bone loss remains unclear.

Current pharmacological treatments for osteoporosis, including antiresorptive and anabolic agents [17], are often associated with significant side effects, especially with long-term use [18]. This underscores the need for alternative therapies with improved safety profiles. Melatonin (N-acetyl-5-methoxytryptamine, MT), a potent endogenous antioxidant and regulator of circadian rhythms, has shown promise in enhancing bone mass and promoting osteogenesis through multiple pathways [19]. Its ability to modulate oxidative stress [20], improve the bone microenvironment [21], and chelate iron [22] makes melatonin an attractive candidate for mitigating ferroptosis and related bone pathologies.

Here, we investigate the impact of chronic SS exposure on bone health and the underlying mechanisms in MC3T3 cells and a mouse model. Our findings reveal that SS induces significant bone loss through ferroptosis activation, which is characterized by increased oxidative stress and impaired osteogenic differentiation. Furthermore, we demonstrate that melatonin effectively mitigates these effects by inhibiting ferroptosis and restoring bone mass and osteogenic potential. These results highlight ferroptosis as a critical mechanism underlying SS-induced osteoporosis and position melatonin as a promising therapeutic agent for its prevention and treatment.

## Materials and methods

### Animal experiments

C57BL/6 mice (male, 19–22 g) were purchased from Jinan Pengyue (Shandong, China). The Animal Ethics Committee of Qingdao University approved all animal study protocols. The mice were all housed in rooms with a 12-h light/dark cycle and had free access to food and water. Every effort was made to minimize the animals' suffering in subsequent treatments. The Food and Agriculture Organization of the United Nations recommends a maximum daily intake of sodium sulfite of 0.7 mg/kg for humans. The administered dose in mice was converted from the human equivalent dose (HED) based on body surface area. Assuming a human body weight of 60 kg, 1000 (mg)/60 (kg) HED = 0.7 × 12.3 = a mouse dose of 8.6 mg/kg [23]. Subsequently, we randomly divided the mice into a control group, a low-dose SS group (8.6 mg/kg), a high-dose SS group (17.2 mg/kg), and a high-dose SS + MT group (MT group; 50 mg/kg), 8 mice in each group. The mice were administered by gavage continuously for 40 days. Finally, the mice were anesthetized and killed, and the femur tissue and blood were collected for subsequent experiments.

### Cell culture and treatment

MC3T3-E1 cells were obtained from Procell Cell Resource Center (Wuhan, China). Cells were cultured in a medium containing α-minimum essential medium (α-MEM; Procell, Wuhan, China) at 37 °C and 5% CO_2_. The medium also contained 10% fetal bovine serum (FBS; Procell, China), 1% 100 μg/mL penicillin, and streptomycin (Procell, China). In subsequent experiments, SS was dissolved in the culture medium. 2 mM SS was used to treat MC3T3-E1 cells for 24 h, after which they were used for transcriptome sequencing. In pharmacological rescue experiments, MT (100 μM) was added to the culture medium for one hour, after which the cells were continued to be treated with 2 mM SS. MC3T3 cells between passages 2 and 6 were used in subsequent experiments.

### Cell viability

MC3T3 cells were homogeneously inoculated in 96-well plates (5000 cells/well), and the cells were treated with different concentrations of SS for 24 h, in drug rescue experiments. After the cells were treated accordingly, the original medium was discarded, and the cells were washed, followed by the addition of 100 μL CCK8 working solution (Solarbio, China) to each well. Finally, the cells were incubated in an incubator for 2 h, and the absorbance at 450 nm was measured using an enzyme marker.

### Osteogenic induction and alkaline phosphatase (ALP) staining

We first treated the cells with medium with or without 2 mM SS for 24 h. When the cell confluence reached 70%, the cells were switched to MC3T3-E1 osteogenic induction and differentiation medium. After 7 days of osteogenic induction of cells, the original medium was discarded and washed twice with PBS. Afterward, they were fixed with 4% paraformaldehyde (PFA) for 20 min at room temperature. ALP staining was subsequently performed using the BCIP/NBT alkaline phosphatase chromogenic kit (Beyotime, China) according to the manufacturer’s instructions. Microscopic images were later obtained using an inverted microscope (Nikon, Japan).

### Alizarin red S (ARS) staining

To assess the degree of mineralization, the Alizarin Red S (ARS) staining kit provided by Beyotime was used for the experiment. Different from the ALP staining, for the ARS staining, the MC3T3 cells needed to undergo osteogenic induction for 21 days. After the induction is completed, the MC3T3 cells were gently rinsed with PBS. Subsequently, the cells are fixed with a 4% paraformaldehyde solution at room temperature. Then, the cells were incubated with the ARS staining solution for 30 min, and finally, images were captured using a microscope.

### Intracellular Reactive Oxygen Species (ROS) measurement

Intracellular ROS production was detected by 2′,7′-dichlorofluorescein diacetate (DCFH-DA) (Beyotime, China). MC3T3 (5000 cells/well) was inoculated in 96-well plates, and the cells were treated with 2 mM SS. In drug rescue experiments, MC3T3 cells were pretreated with MT (100 μM) for 1 h before adding 2 mM SS for treatment. Before staining, cells were washed with serum-free medium, and the indicated concentrations of DCFH-DA probe were added according to the instructions and incubated at 37 °C for 30 min. Finally, the fluorescence intensity of ROS was observed with a fluorescence microscope (Nikon, Japan).

### C11-BODIPY and FerroOrange staining

MC3T3 cells were inoculated in 24-well plates at a density of 2 × 10^5^ per well, and after treatment with the corresponding drugs, the cells were incubated with the working solution of C11-BODIPY 581/591 or FerroOrange working solution in an incubator for 30 min. Images were captured by fluorescence microscopy. When lipid peroxidation occurred, the red fluorescence changed to green fluorescence. FerroOrange probe (Dojindo, China) was used to detect the intracellular Fe^2+^ content.

### Measurement of glutathione (GSH) and malondialdehyde (MDA)

MDA and GSH levels in mouse serum were measured using the MDA Assay Kit (servicebio, China) and GSH Assay Kit (Servicebio, China), respectively, according to the manufacturer's instructions.

### Quantitative real-time PCR (qPCR) analysis

Briefly, total RNA was extracted from treated cells using the FastPure Complex Tissue/Cell Total RNA Isolation Kit (Vazyme, China), and cDNA was obtained by reverse transcription using the Reverse Transcription Kit (Vazyme, China). cDNA was subsequently amplified by a qPCR detection system (Thermo Fisher Scientific, USA) with β-actin as the internal reference gene. The primers and primer sequences used for qPCR analysis are listed in Table 1.

### Micro-computed tomography (micro-CT)

The right femur of mice was fixed in 4% PFA for 24 h and then scanned using a Micro-CT system (PerkinElmer, Japan) at a voxel size of 36 μm, a voltage of 50 kV, and a current of 100 μA settings. The region of interest for analyzing bone morphometric parameters was defined as starting from the end of the growth plate of the distal femur and extending until 1 mm in the direction away from the metaphysis. The parameters we were interested in included bone volume versus tissue volume (BV/TV), trabecular number (Tb.N), trabecular thickness (Tb.Th), and trabecular separation (Tb.Sp).

### Western blot (WB) assay

Cells and mouse femurs were lysed with a RIPA (Radio Immunoprecipitation Assay Lysis Buffer) containing protease and phosphatase inhibitors, followed by protein quantification using a BCA kit. Aliquots of protein samples from different groups were electrophoresed on 10% sodium dodecyl sulfate polyacrylamide gels and transferred to PVDF membranes. Milk was used to block the membrane for 1 h and then washed three times with TBST, and the last was added with the corresponding primary antibody and incubated overnight at 4 °C. The next day, the membrane was washed three times with TBST and incubated with the corresponding secondary antibody for 1 h at room temperature, and finally, chemiluminescence was performed (Viber, France). The antibodies used in this study were listed below: GPX4 (Affinity, DF6701, 1:1000), Slc7a11 (Boster, ACGI-19, 1:1000), FTH1 (Santa Cruz, sc-376594, 1:500), β-actin (Proteintech, 81115-1-RR, 1:20,000), mouse secondary antibodies (1:5000; Boster; BA1050) and rabbit secondary antibodies (1:5000; Boster; BA1054).

### Hematoxylin–eosin (HE) staining

Mouse femurs were first fixed and decalcified, followed by tissue dehydration and paraffin embedding. Sections were cut into 4-μm-thick and later stained according to the HE kit instructions. Finally, the sections were sealed with neutral resin. We captured the images with a slide scanner (3DHISTECH, Hungary).

### Immunohistochemical (IHC) analysis

4-μm-thick sections were deparaffinized, hydrated, and antigenically repaired sequentially following the steps. Endogenous peroxidase activity was blocked with hydrogen peroxide at room temperature, and the sections were washed three times with PBS and later closed with 5% goat serum. Sections were incubated overnight in the appropriate primary antibody. Sections and SABC-HRP kits containing anti-rabbit IgG (Beyotime, China) and anti-mouse IgG (Beyotime, China) were incubated at 37 °C. The sections were incubated with DAB Horseradish Peroxidase (Beyotime, China). The color immunohistochemical (IHC) analysis development was terminated at the appropriate time, and the images were re-stained with hematoxylin and finally captured with a slide scanner (3DHISTECH, Hungary).

### Statistical analysis

All experiments were repeated at least three times. Data were statistically analyzed using GraphPad Prism 10.0. The data are presented as the mean ± standard deviation (mean ± SD). The Shapiro–Wilk test was employed to assess the normality of the data distribution. In cases where the data conformed to a normal distribution, Student’s t-test or one-way analysis of variance (ANOVA) were utilized to analyze and compare differences among groups. Conversely, for data sets that deviated from normality, a non-parametric approach, specifically the Kruskal–Wallis test, was implemented to evaluate group disparities. *p*-values < 0.05 were considered statistically significant.

## Results

### Sodium sulfite exposure resulted in bone loss in mice

**Fig. 1:**
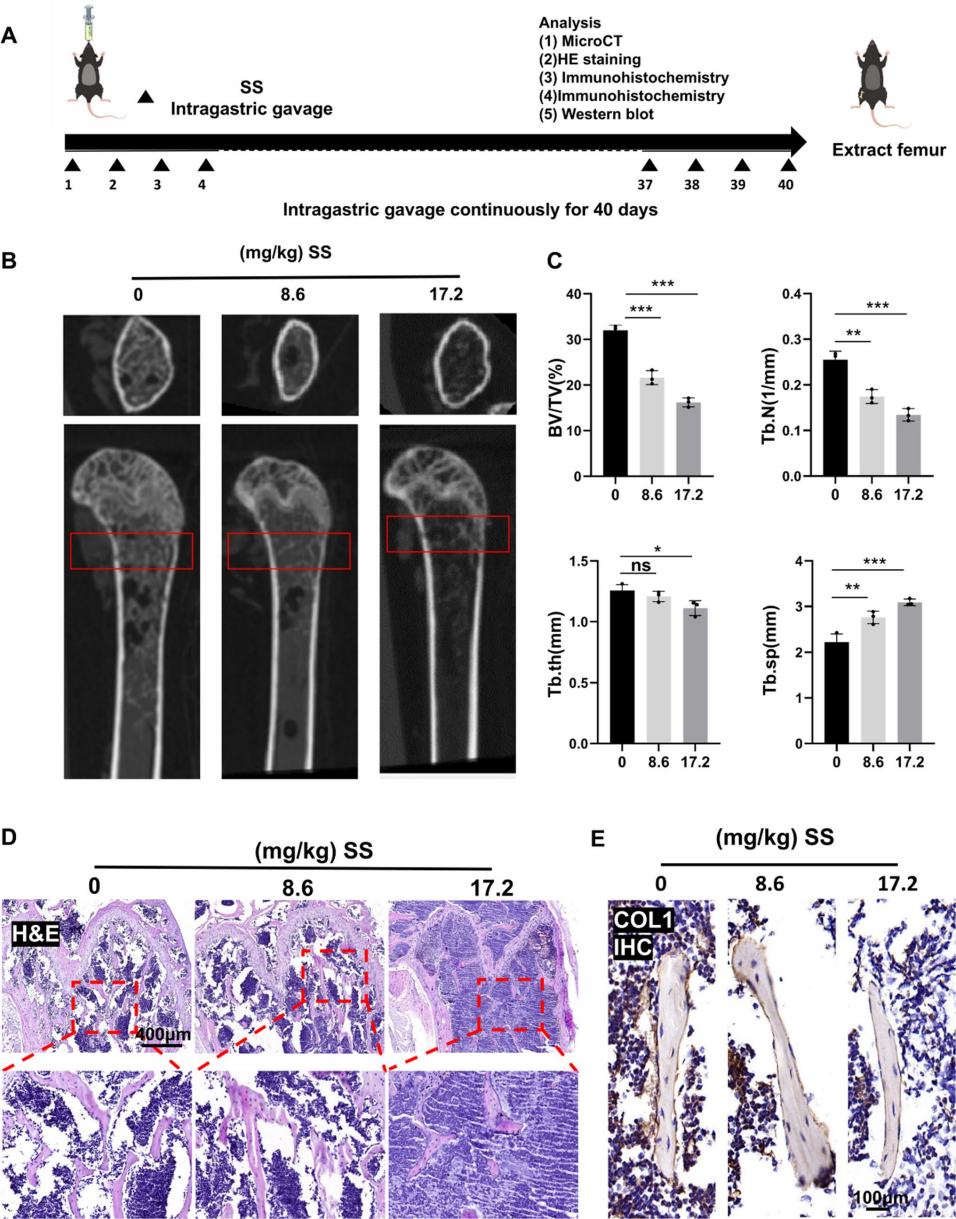
Sodium sulfite-induced bone loss in mice. **A** Patterns of sodium sulfite gavage for 40 days in C57BL/6 mice (0, 8.6, and 17.2 mg/kg). **B**, **C** Representative horizontal and sagittal cross-sectional images of the distal femur Micro-CT in mice with its region of interest (red box) and corresponding bone morphological parameters analysis (n = 3). **D** Representative images of HE staining of the distal femur in control and mice exposed to different doses of SS (70×, Scale bar, 400 μm, n = 3). **E** Representative images of Col-1 IHC staining in control and SS-exposed mice (400×, Scale bar, 100 μm, n = 3). Values are shown as mean ± SD. *p < 0.05, **p < 0.01, ***p < 0.001, ns, not significant

As shown in Fig. 1A, we exposed C57BL/6 mice to SS (0, 8.6 and 17.2 mg/kg) by continuous gavage for 40 days to evaluate the effect of sodium sulfite on bone mass. The micro-CT results showed that SS had reduced bone mass in a dose-dependent manner compared with controls (Fig. 1B). Analysis of bone morphometric parameters showed that BV/TV and Tb.N decreased and Tb.Sp increased in the distal femur of mice.Tb.th decreased in the group exposed to the low-dose SS, but the decrease was not statistically significant and showed a significant decrease in the group exposed to the high-dose SS (Fig. 1C). We suspected that it might be that the thinner bone trabeculae were reduced first and the thicker ones had not yet changed, eventually leading to little change in thickness. HE staining result consistent with the above results. We were able to observe a decrease in the number of bone trabeculae and an increase in the separation rate (Fig. 1D). The above suggested that SS exposure can successfully model reduced bone mass in mice. Finally, we performed immunohistochemical staining of type I collagen (Col-1) to investigate the effect of SS on osteogenesis. The results showed that the number of osteoblasts expressing Col-1 was reduced after SS exposure, and the relative mean optical density value of Col-1 was decreased in a dose-dependent manner by SS (Fig. 1E and FigSI. 1A). This suggests that prolonged SS exposure affects the osteogenic capacity of mice.

Sodium sulfite exposure resulted in diminished osteogenic capacity and the induction of ferroptosis in MC3T3 cells.

**Fig. 2:**
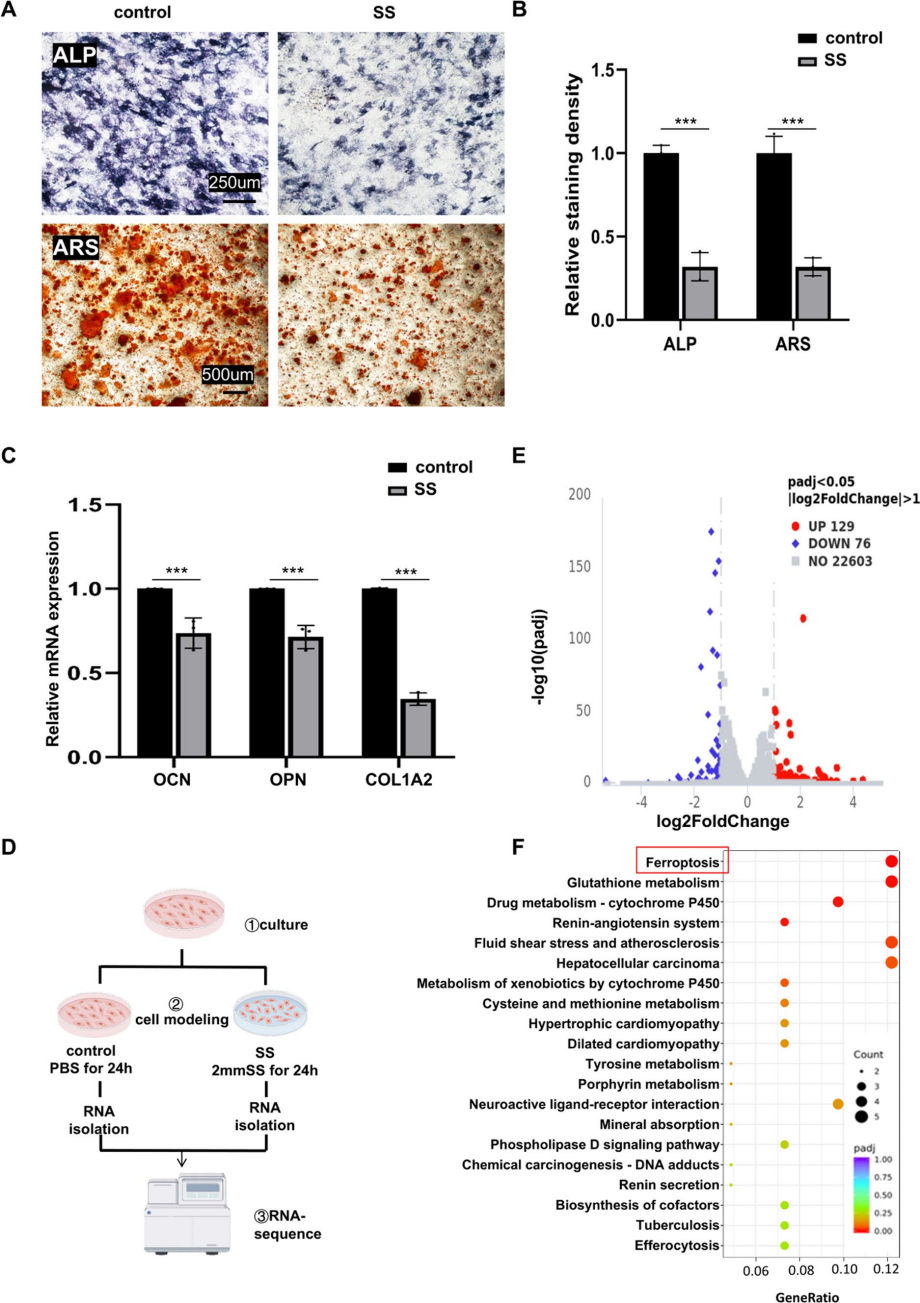
SS led to decreased osteogenic capacity and ferroptosis in MC3T3. **A** Representative images of ALP(100×, Scale bar, 250 μm, n = 3) and ARS(40×, Scale bar, 500 μm, n = 3). **B** Quantitative analysis of ALP and ARS. **C** The expression of osteogenic-related genes (OCN, OPN, and Col1A2) was analyzed by qPCR in control and SS-exposed groups (n = 3). **D** Diagram of sample preparation and experimental design for transcriptome analysis. **E**, **F** Volcano plots showing differential genes in the control and SS groups. Red represents up-regulated genes and blue represents down-regulated genes. KEGG enrichment of differential genes was performed for the top 20 pathways enriched

First, we co-incubated MC3T3 cells with different concentrations of SS for 24 h to clarify the effect of SS on MC3T3 cell viability. When the concentration reached 2 mM, the cell viability decreased significantly (FigSI. 1B). If the cells were treated with higher concentrations of SS, we could not collect enough cells for the experiment. Therefore, we chose to treat MC3T3 cells with 2 mM SS for 24 h for subsequent experiments. To understand whether the osteogenic differentiation capacity of MC3T3 was affected by SS exposure, we subjected cells with or without SS treatment for 24 h to osteogenic differentiation induction. After 7 days, ALP staining and real-time quantitative polymerase chain reaction (qPCR) analysis were performed. ARS staining was performed 21 days after osteogenic induction. ALP and ARS staining results showed a decrease in the positive areas of staining in SS-treated cells (Fig. 2A, B). The qPCR results showed that the expression of osteogenesis-related genes (OCN, OPN, and COL1A2) in the SS treatment group cells was down-regulated (Fig. 2C). The above results suggested that SS stimulation impaired the osteogenic capacity of MC3T3. The results showed that in agreement with the in vivo experiments, SS exposure impaired the osteogenic capacity of MC3T3 cells. To further explore the molecular mechanisms by which SS induces MC3T3 cell death and reduced osteogenic capacity, we performed RNA sequencing to identify differential genes in SS-exposed and control groups. A schematic diagram of the transcriptome sequencing preparation is shown in Fig. 2D. Twenty-four hours after treating cells with SS, the transcriptome results showed 205 differentially expressed genes (*p* value < 0.05, fold change > 2) (Fig. 2E). Next, we performed enrichment analysis based on differential genes. The results showed that the ferroptosis pathway was significantly enriched (Fig. 2F).

**Fig. 3:**
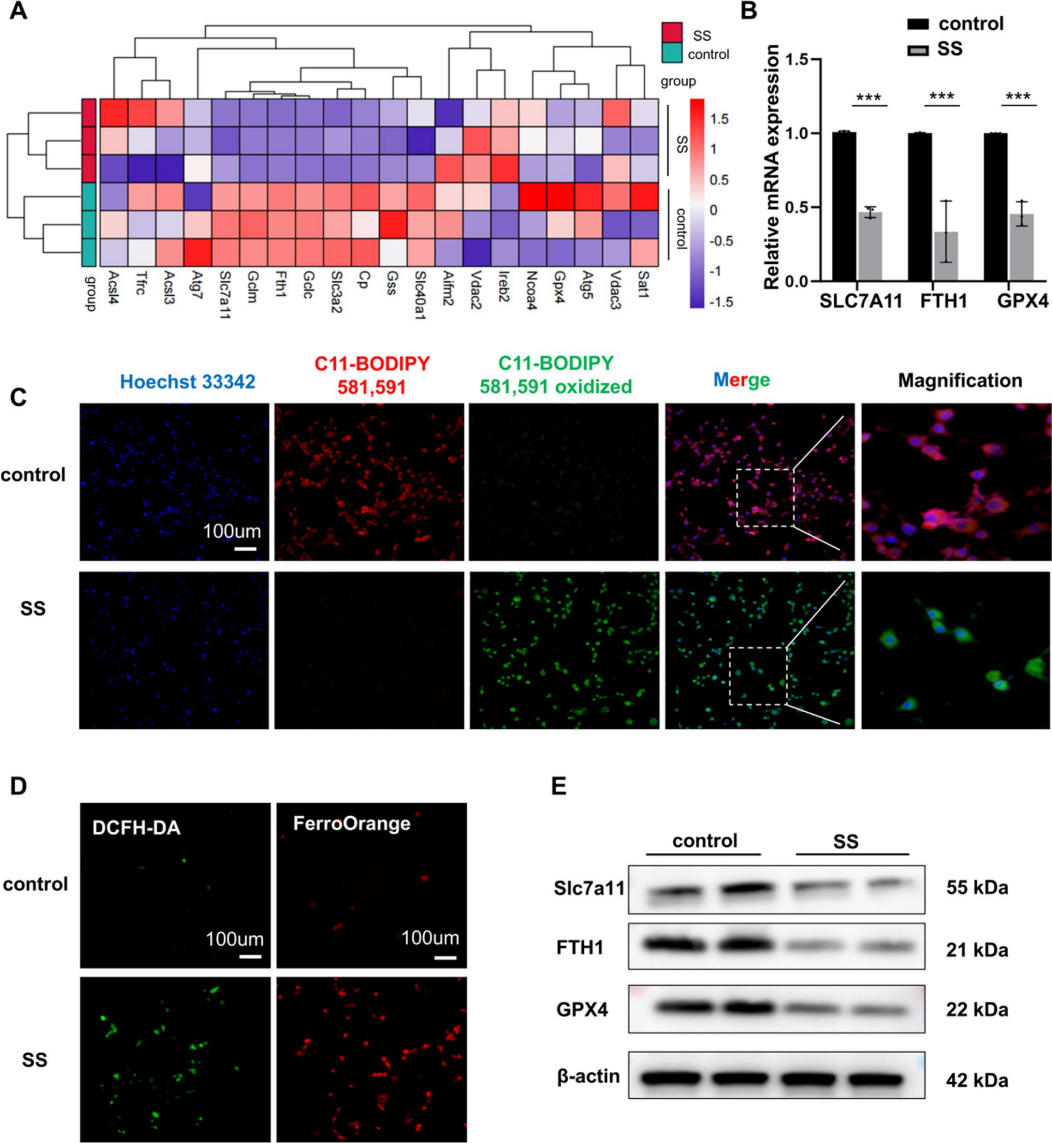
SS led to decreased osteogenic capacity and ferroptosis in MC3T3. **A** Heatmap of 20 differentially expressed ferroptosis-related genes. **B** qPCR was performed to verify the content of SLC7A11, FTH1, and GPX4 in the control and SS groups (n = 3). **C** Representative images of C11-BODIPY 581/591 staining and data analysis. (200×, Scale bar, 100 µm, n = 3). **D** Representative stained images of DCFH-DA and FerroOrange of MC3T3 (200×, scale bar, 100 μm, n = 3). **E** WB showing the expression levels of SLC7A11, FTH1, and GPX4 in MC3T3 treated with SS for 24 h (n = 3). Values are shown as mean ± SD. **p < 0.01, ***p < 0.001

We then mapped some of the ferroptosis-related genes into a heat map based on the sequencing results (Fig. 3A), and then we used qPCR to validate the RNA sequencing results. The results showed that the mRNA content of ferroptosis-related genes, including Slc7A11, FTH1, and GPX4 was reduced (Fig. 3B). Therefore, we hypothesized that SS might induce ferroptosis in MC3T3 cells. Ferroptosis is generally accompanied by a series of changes, increased Fe^2+^ content, reactive oxygen species production, and lipid peroxidation [18]. To further confirm the occurrence of ferroptosis, we performed C11-BODIPY staining, DCFH-DA staining, and FerroOrange staining in MC3T3 with or without SS exposure. In the SS-exposed group, C11-BODIPY produced bright green fluorescence, indicating lipid peroxidation (Fig. 3C and FigSI. 1C); DCFH-DA staining showed increased bright green fluorescence; the fluorescence intensity of the FerroOrange probe surged (Fig. 3D and FigSI. 1D, E). Finally, the results of the Western Blot assay for Slc7A11, FTH1, and GPX4 were consistent with the previous results. Those were, the expression of these three proteins decreased under SS stimulation (Fig. 3E and FigSI. 1F). It indicated that SS was able to activate MC3T3 cells to undergo ferroptosis. We hypothesized that SS may impair its osteogenic capacity by inducing MC3T3 to activate ferroptosis.

### Sodium sulfite exposure induced ferroptosis in mice

**Fig. 4:**
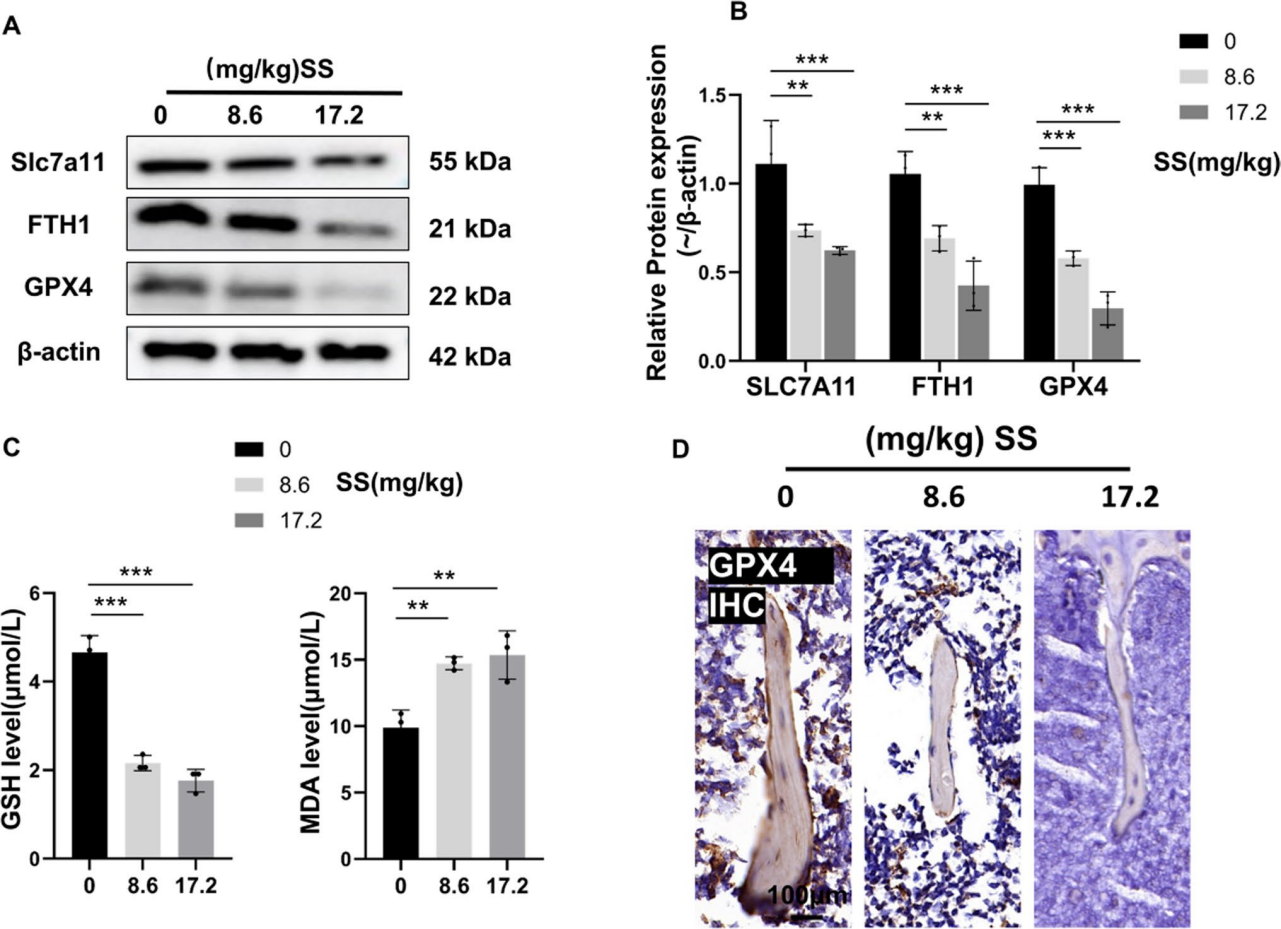
SS-induced ferroptosis in mice. **A**, **B** Protein expression of SLC7A11, FTH1, and GPX4 and their quantification in mice femur after 40 days of SS (0, 8.6, and 17.2 mg/kg) gavage (n = 6). **C** GSH and MDA levels were determined by the kit (n = 3). **D** Representative images of IHC staining of GPX4 in the distal femur of control and SS-exposed mice (400×, Scale bar, 100um, n = 3). Values are shown as mean ± SD. **p < 0.01, ***p < 0.001

Subsequently, to further analyze the expression levels of ferroptosis-related proteins in mouse femurs, we performed WB experiments. WB detected the protein levels of Slc7A11, FTH1, and GPX4. The results showed that exposure to SS inhibited the protein expression of Slc7A11, FTH1, and GPX4, and the degree of decrease was positively correlated with the dose of SS (Fig. 4A, B). This is consistent with the sequencing results of the cells, suggesting that exposure to SS activates ferroptosis. We then further analyzed the serum levels of GSH and MDA in mice and found that exposure to SS decreased GSH levels but increased MDA levels (Fig. 4C). Immunohistochemistry for GPX4 showed a reduction in positive areas for GPX4 in the low- and high-dose SS exposure groups compared to the control group (Fig. 4D). All the above suggested that SS can also activate ferroptosis in vivo.

Melatonin attenuated SS-induced ferroptosis in MC3T3 cells and partially restored their osteogenic differentiation capacity.

**Fig. 5:**
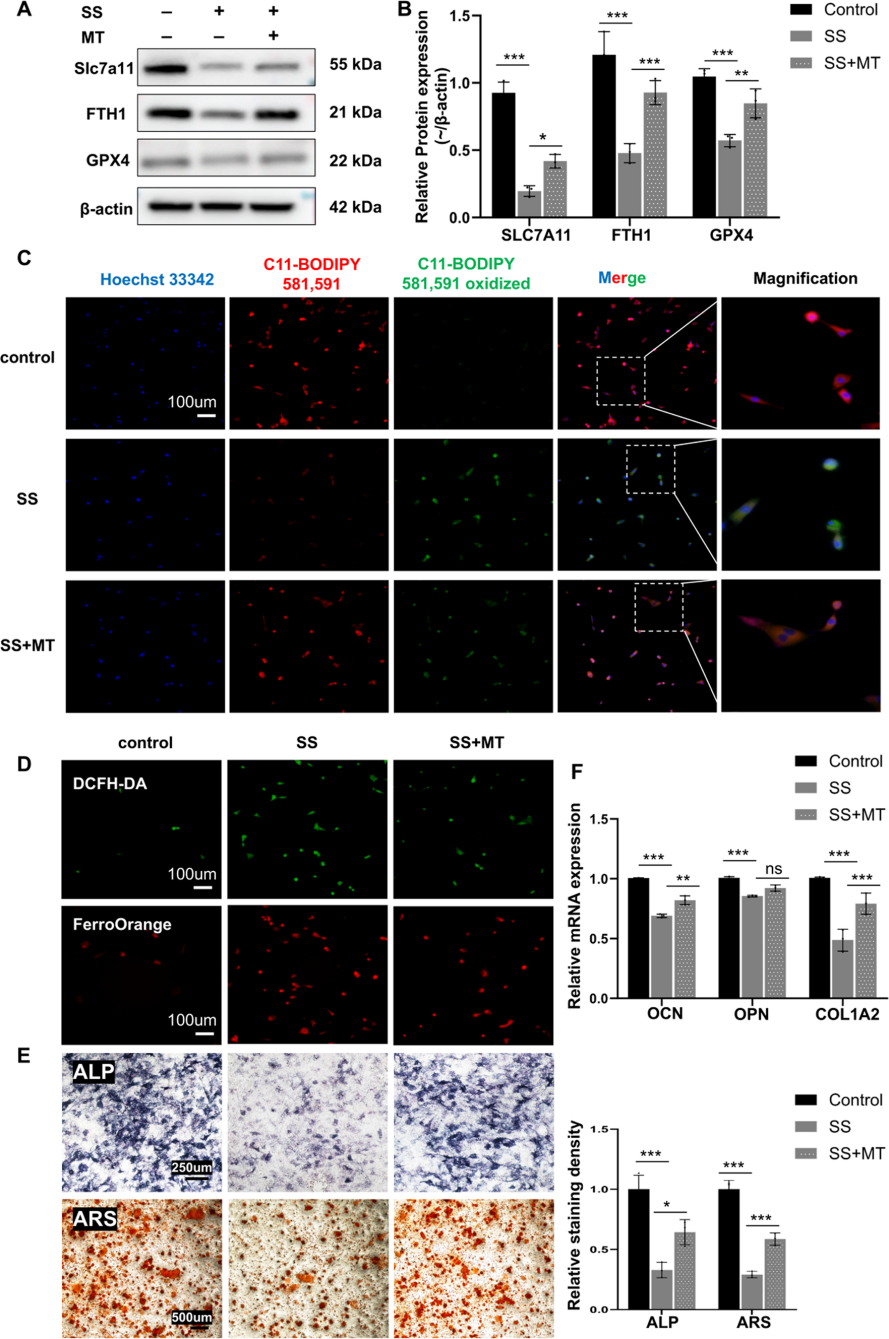
Melatonin attenuated SS-induced MC3T3 ferroptosis and partially restored osteogenic differentiation capacity. **A**, **B** SLC7A11, FTH1, and GPX4 protein expression and quantification (n = 3). **C** Representative images of C11-BODIPY 581/591 staining (200×, Scale bar, 100 µm, n = 3). (**D**) Representative images of DCFH-DA and FerroOrange staining (200×, Scale bar, 100 µm, n = 3). (**E**) Representative images of ALP staining (100×, Scale bar, 250 µm, n = 3) and ARS staining (40×, Scale bar, 500 µm, n = 3). (**F**) OCN, OPN, and COL1A2 were measured by qPCR (n = 3). Values are shown as mean ± SD. *p < 0.05, **p < 0.01, ***p < 0.001, ns, not significant

We chose MT to treat SS-induced MC3T3 ferroptosis and reduced osteogenic capacity. We divided the cells into three groups: (1) control group; (2) SS group; and (3) SS + MT group. Western Blot results showed that MT partially restored the low expression levels of GPX4, Slc7A11, and FTH1 caused by SS stimulation (Fig. 5A, B). Therefore, we speculated that MT could attenuate SS exposure-induced ferroptosis in osteoblasts. To further prove this speculation, we performed C11-BODIPY staining, DCFH-DA staining, and FerroOrange staining on three groups of cells. Consistent with the WB results, MT significantly improved the SS stimulation-induced changes in MC3T3 cell-associated probe staining (Fig. 5C, D and FigSI. 2A, B, C). Based on the above information, we speculated that MT could ameliorate the decreased osteogenic capacity of MC3T3 cells by inhibiting ferroptosis. Subsequently, ALP and ARS staining was performed on the three groups of cells, and the results showed that the ALP and ARS staining area was larger in the MT-treated group than in the SS group, which implied that MT could improve the osteogenic capacity (Fig. 5E). For further verification, we detected the levels of OCN, OPN, and COL1A2 mRNA in MC3T3 cells by RT-PCR. Compared with SS stimulation alone, pretreatment with MT up-regulated OCN and COL1A2 gene expression levels, and OPN was also up-regulated but without significant difference (Fig. 5F). In conclusion, MT inhibited MC3T3 ferroptosis and ameliorated the decrease in osteogenic capacity induced by SS.

### Melatonin attenuated SS-induced bone loss in mice

**Fig. 6:**
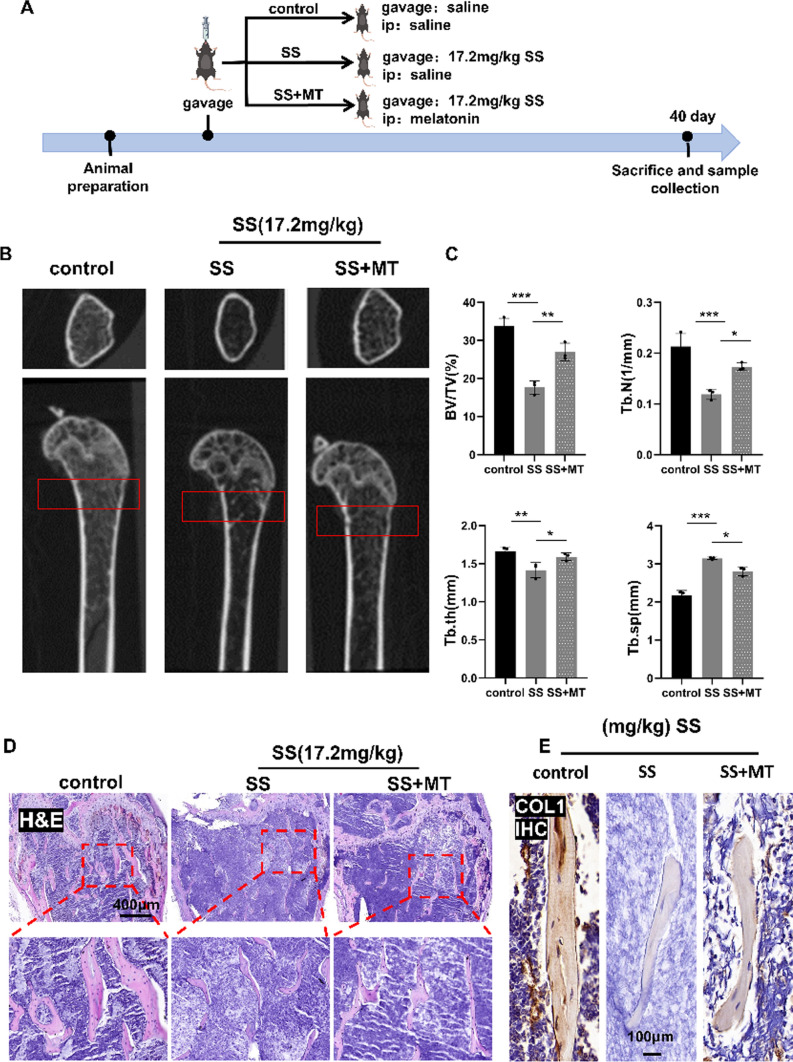

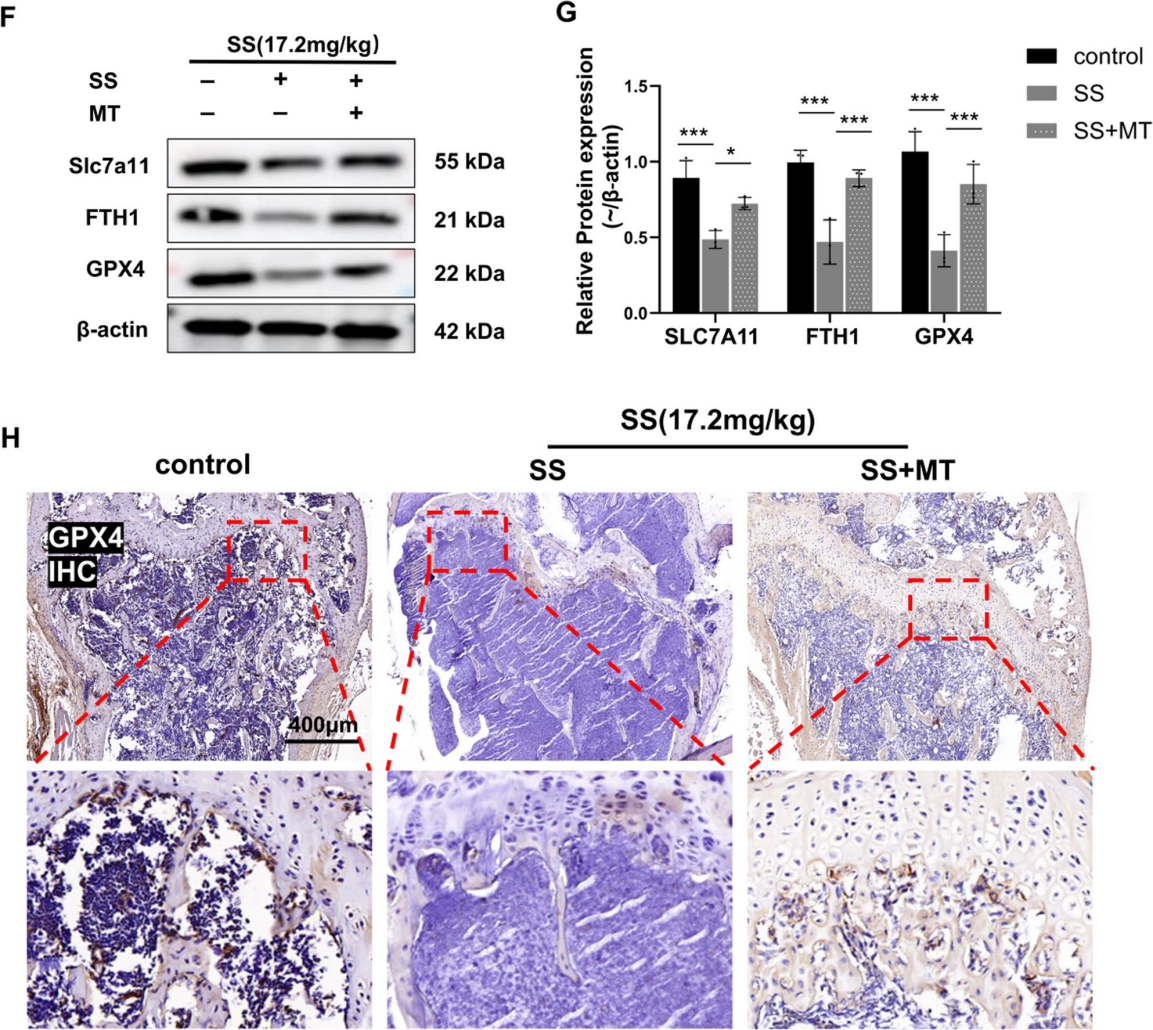
Melatonin attenuates SS-induced bone loss in mice. **A** Schematic diagram showing the course of drug administration in different groups of mice. **B**, **C** Representative images of Micro-CT of the distal femur in different groups of mice and analysis of their associated bone morphometric parameters (n = 3). **D** Representative images of HE staining (70×, Scale bar, 400 µm, n = 3). **E** Representative images of col-1 IHC staining of mice distal femur (400×, Scale bar, 100 µm, n = 3). **F**, **G** Protein expression levels of SLC7A11, FTH1, and GPX4 in mice femur and related quantitative analyses (n = 6). **H** Representative images of IHC staining of GPX4 in the distal femur (70×, Scale bar, 400 µm, n = 3). Values are shown as mean ± SD. *p < 0.05, **p < 0.01, ***p < 0.001

Subsequently, we examined the potential of MT to attenuate bone loss induced by SS in mice. The mice were categorized into three distinct groups: (1) control group; (2) SS group (SS 17.2 mg/kg); and (3) SS + MT group (SS 17.2 mg/kg, MT 50 mg/kg). The mice were anesthetized and euthanized 40 days following the administration of SS, after which the tissues were collected for subsequent experiments (Fig. 6A). Micro-CT analysis revealed that the SS-treated mice exhibited reduced bone volume, fewer trabecular structures, decreased thickness, and elevated separation rates compared to both the control and MT treatment groups. These findings were statistically significant, with the bone microstructural parameters of the MT-treated group falling between those of the control and SS groups (Fig. 6B, C). Histological examination via HE staining corroborated these results, demonstrating smaller spacing between bone trabeculae in the MT-treated group relative to the SS group (Fig. 6D). We sought to determine whether MT could also enhance osteogenesis in vivo. IHC staining revealed an increase in Col-1-positive cells post-MT treatment, indicating a rise in Col-1-expressing osteoblasts within treated mice (Fig. 6E and FigSI. 2D). We concluded that MT mitigated bone loss in mice to some extent. To further investigate whether the inhibitory effect of MT on SS-induced bone loss in mice is related to ferroptosis, we examined the serum levels of GSH and MDA and carried out Western Blot and IHC experiments. Our results demonstrated that MT significantly upregulated GSH levels while reducing MDA levels when compared to SS group (FigSI. 2E, F). WB results indicated an increase in protein expression levels of GPX4, SLC7A11, and FTH1 in the MT-treated group compared to the SS group (Fig. 6F, G). IHC staining for GPX4 aligned with these protein expression findings following SS stimulation; specifically, there was a decrease in relative mean optical density values for GPX4 that was partially restored by MT treatment. This suggests that MT may inhibit ferroptosis induced by SS (Fig. 6H, FigSI. 2G).

## Discussion

Our study provides compelling evidence that chronic exposure to sodium sulfite induces significant bone loss in mice and activates ferroptosis in MC3T3 cells. Using in vivo and in vitro models, we demonstrate that sodium sulfite disrupts bone homeostasis by impairing osteoblast function through the ferroptosis pathway, a novel mechanism in the pathogenesis of osteoporosis. Furthermore, we show that melatonin, a potent antioxidant and iron chelator, effectively mitigates these adverse effects, restoresbone mass and osteogenic capacity by inhibiting ferroptosis.

Sodium sulfite, widely used as a food additive, has been associated with various adverse health effects, particularly with chronic or excessive consumption [23, 25]. Despite its widespread use, its potential impact on bone health has been underexplored to date. Here, we established a murine model of sodium sulfite-induced osteoporosis through prolonged gavage and corroborated these findings with cellular models of osteogenesis. RNA sequencing and subsequent biochemical analyses revealed that SS exposure significantly activates ferroptosis-related pathways, including decreased expression of GPX4, SLC7A11, and FTH1, along with increases oxidative stress markers. These results highlight ferroptosis as a key mechanism underpinning sodium sulfite-induced bone loss.

Ferroptosis, characterized by iron accumulation and lipid peroxidation [26], has recently been implicated in various degenerative diseases, including bone disorders such as osteoarthritis [27] and glucocorticoid-induced osteoporosis [28]. Our findings extend this paradigm by identifying ferroptosis as a critical contributor to sodium sulfite-induced osteoporosis. The accumulation of ROS and diminished antioxidant defenses disrupt the osteogenic capacity of MC3T3 cells, resulting in reduced expression of osteogenesis-related markers and impaired differentiation. These findings establish a direct link between ferroptosis and osteoblast dysfunction in the context of sodium sulfite exposure.

Melatonin emerged as an effective therapeutic candidate in our study, mitigating the adverse effects of sodium sulfite both in vivo and in vitro. As an endogenous hormone with diverse physiological roles, melatonin exhibits strong antioxidant properties [29, 30], modulates circadian rhythms [31], and regulates bone metabolism [32–34]. Notably, melatonin enhanced the expression of ferroptosis regulators, including GPX4 and SLC7A11, and restored cellular antioxidant capacity, reducing lipid peroxidation and ROS levels. These effects translated to improved osteogenic differentiation in vitro and partial recovery of bone mass in vivo. Additionally, melatonin’s ability to reduce the RANKL/OPG ratio further supports its role in counteracting bone resorption [35, 36], making it a promising alternative to current osteoporosis treatments.

Existing pharmacological agents for osteoporosis, such as antiresorptive drugs and anabolic therapies like teriparatide, are limited by their side effects, high costs, and administration challenges [37]. Melatonin’s favorable safety profile, diverse modes of administration [38], and dual capacity to inhibit bone resorption and promote osteogenesis [39] present a significant advantage. Its iron-chelating ability further underscores its potential for targeting ferroptosis-driven bone pathologies, and it offers a novel therapeutic approach for conditions previously refractory to treatment.

In conclusion, our study provides the first evidence linking sodium sulfite exposure to ferroptosis-mediated osteoporosis, and it demonstrates the therapeutic potential of melatonin in mitigating this process. By addressing both the underlying mechanisms and potential interventions, our findings advance our understanding of sodium sulfite-induced bone pathologies, paving the way for innovative strategies in osteoporosis management.

## Supplementary Information

Below is the link to the electronic supplementary material.

Supplementary file1 (DOCX 1799 KB)

## Data Availability

The data used to support the findings of this study are available from the corresponding author upon reasonable request.
