# Supplementary material for: Melatonin alleviates sodium sulfite-induced osteoporosis in mice via suppression of the ferroptosis pathway
Source: Apoptosis. 2025 Jul 18;30(9-10):2301–15. doi: 10.1007/s10495-025-02135-8 (PMC12474642; doi:10.1007/s10495-025-02135-8)
Supplement: Supplementary file 1 — Supplementary file1 (DOCX 1799 KB) [file 10495_2025_2135_MOESM1_ESM.docx]

**Sodium sulfite induces osteoporosis in mice via ferroptosis**

Qiuping He^1*^, Lei Xie^1*^, Haining Peng^1^, Xiao Xiao^2, ##^, Tengbo Yu^3, #^

1. Department of Orthopedic Surgery, Qingdao Municipal Hospital, Qingdao University, Qingdao, China.

2. Central Laboratories, Qingdao Municipal Hospital, University of Health and Rehabilitation Sciences, Qingdao, China

3. Department of Orthopedic Surgery, Qingdao Municipal Hospital, University of Health and Rehabilitation Sciences, Qingdao, China

#Correspondence: yutengbo.dr@hotmail.com (Tengbo Yu)

##Correspondence: 1185958508@qq.com (Xiao Xiao)

*These authors contributed equally to this work.

**Supplementary Material：**

**
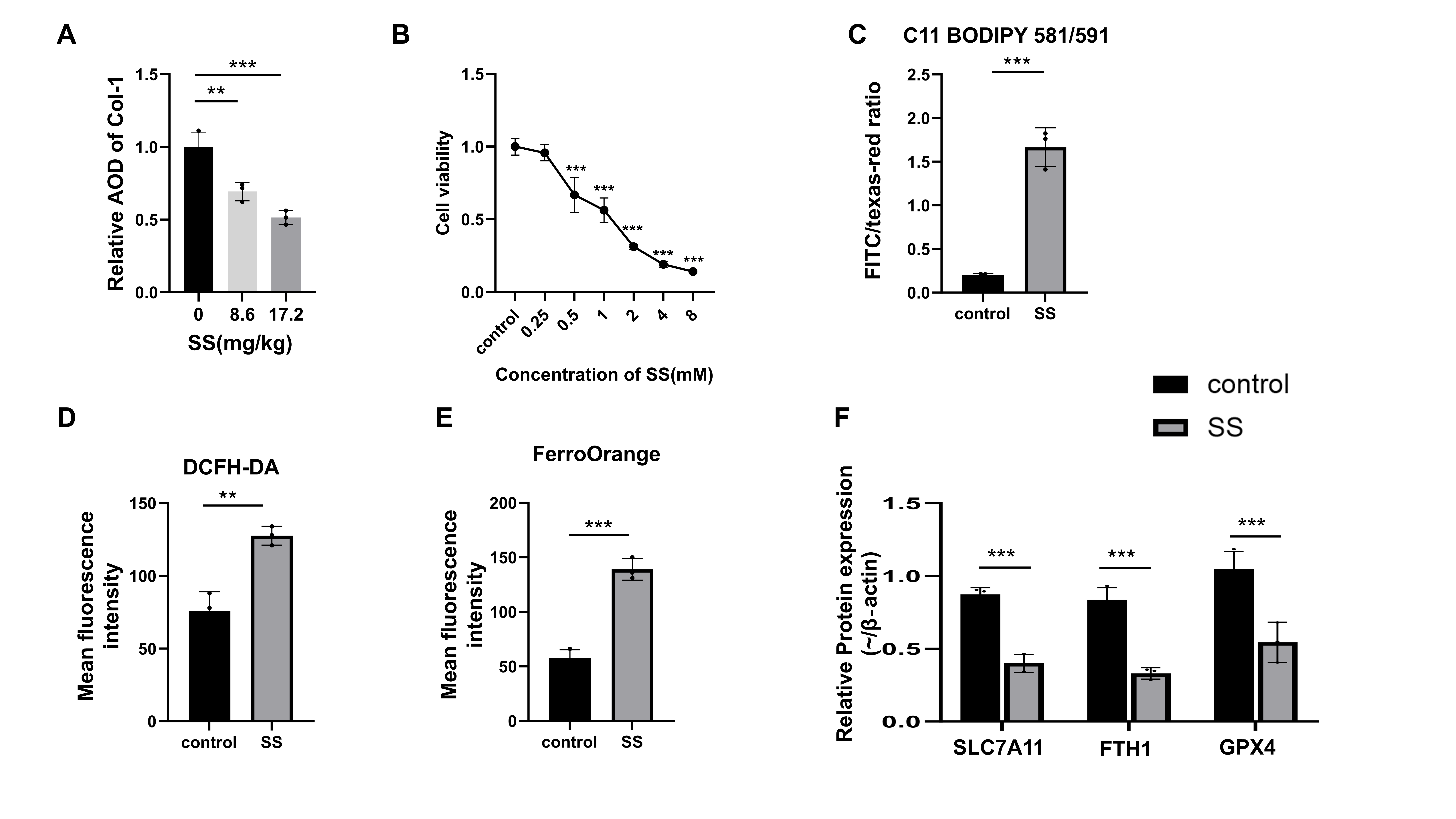
**

*Fig.S1. (A) Quantitative analysis of Col-1 relative average optical density (AOD) results (n=3). (B) Effect of different concentrations of SS (0.25, 0.5, 1, 2, 4 or 8 mm) for 24 h on MC3T3 cell viability. (C) Quantitative analysis of C11 BODIPY fluorescence (n=3). (D) Quantitative analysis of DCFH-DA fluorescence (n=3). (E) Quantitative analysis of FerroOrange fluorescence (n=3). (F) Quantitative analysis of ferroptosis-related genes (SLC7A11, FTH1, GPX4) was conducted in the control group and the SS-exposed group (n = 3). Values are shown as mean±SD. **p<0.01, ***p<0.001.*

**
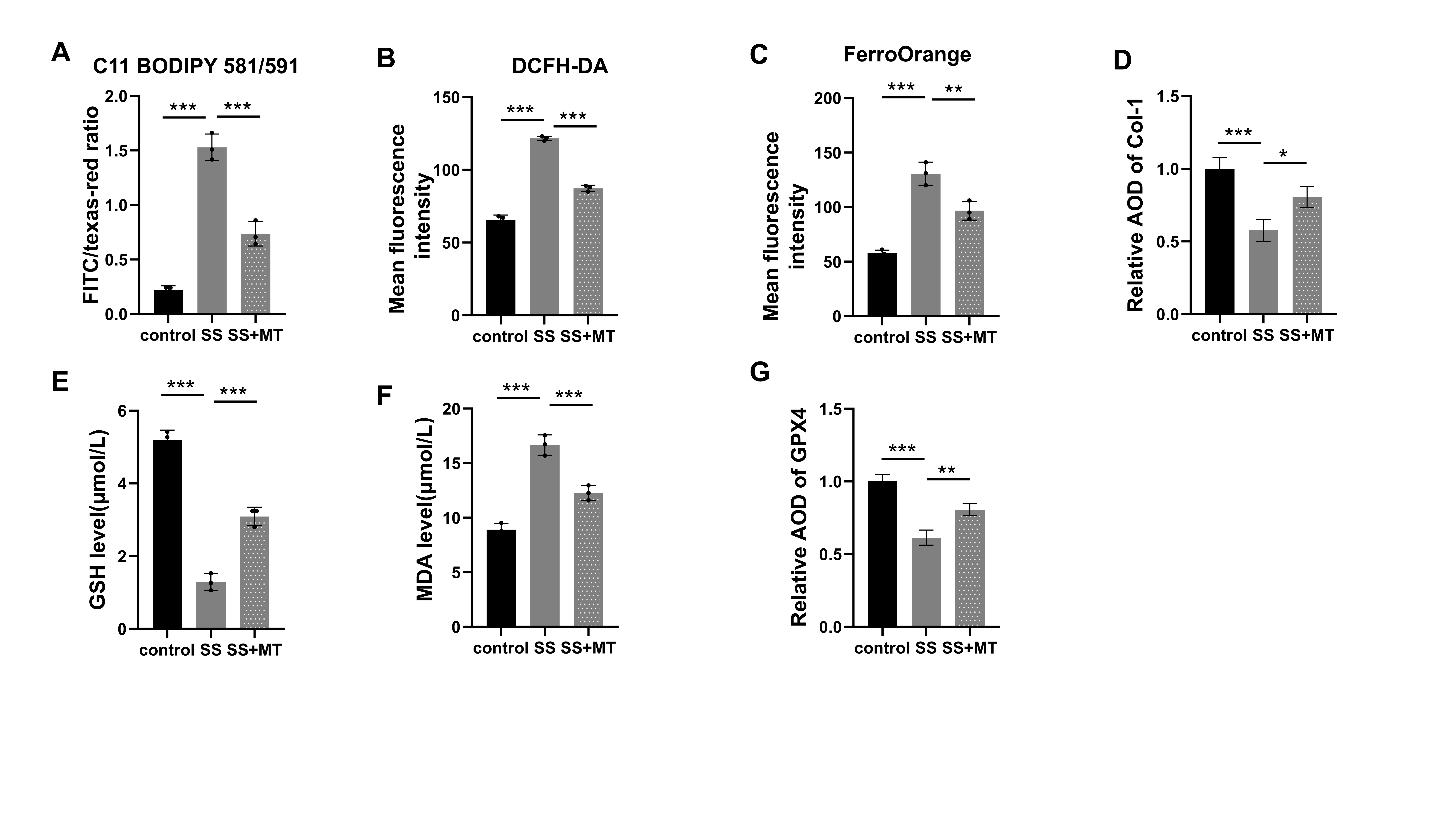
**

*Fig.S2. (A) Quantitative analysis of C11 BODIPY fluorescence (n=3). (B) Quantitative analysis of DCFH-DA fluorescence (n=3). (C) Quantitative analysis of FerroOrange fluorescence (n=3). (D) Quantitative analysis of Col-1 relative average optical density (AOD) results (n=3). (E, F) GSH and MDA levels in mouse serum (n=3). (G) Quantitative analysis of GPX4 relative average optical density (AOD) results (n=3).*

Table S1 Primers used for qPCR.

|  | **Forward primer sequence (5'-3')** | **Reverse primer sequence (5'-3')** |
| --- | --- | --- |
| **β-actin** | CATCCGTAAAGACCTCTATGCCAAC | ATGGAGCCACCGATCCACA |
| **GPX4** | ATAAGAACGGCTGCGTGGTGAAG | TAGAGATAGCACGGCAGGTCCTTC |
| **SLC7A11** | CTTTCAAGGTGCCTCTCTTCATCCC | GGTGCTGAATGGGTCCGAGTAAAG |
| **FTH1** | TGCCATCAACCGCCAGATCAAC | AAGTTCTTCAGAGCCACATCATCTCG |
| **OPN** | CCATCTCAGAAGCAGAATCTCCTTG | CTCCATCGTCATCATCATCGTCATC |
| **OCN** | AGCAGGAGGGCAATAAGGTAGTG | CTCGTCACAAGCAGGGTTAAGC |
| **COL1A2** | CCGAGGCAGAGATGGTGTTGATG | TAGGCAGCAAAGTTCCCAGTAAGAC |
